# Orthodontic management of severe inversely impacted maxillary central incisors: a case series

**DOI:** 10.3389/froh.2024.1474190

**Published:** 2024-10-16

**Authors:** Yuriko Maruya, Ryoko Hino, Manami Tadano, Seira Hoshikawa, Shinji Otake, Yuta Chiba, Kan Saito

**Affiliations:** Division of Pediatric Dentistry, Department of Oral Health and Development Sciences, Tohoku University Graduate School of Dentistry, Sendai, Japan

**Keywords:** dilaceration, maxillary central incisor, inversely impacted, orthodontic, fenestration, orthodontic traction, case report

## Abstract

**Background:**

Abnormal positioning and dislocation of the central incisor can disturb tooth eruption. Generally, inversely impacted maxillary central incisors do not erupt naturally. Performing traction and applied extrusion of an inversely impacted maxillary central incisor with a high inclination angle of the crown is challenging. This study aimed to examine the possibility of orthodontic treatment for severely inversely impacted maxillary central incisors in a series of case studies.

**Methods:**

The inclination angle of the tooth crown, curvature of the tooth root, and length of the formed tooth root were measured using radiography. The teeth were then fenestrated and traction was applied using a lingual arch appliance with elastics.

**Results:**

The average crown axis inclination was 113°, the degree of root curvature was 97.3°, and the root formation was 36.1%. Although the crown axis inclination and root curvature were severe, all the incisors were aligned in the correct position as vital teeth through surgical and orthodontic treatments.

**Conclusions:**

Traction should be performed in the early period of incisor development when root formation is not progressing, regardless of the tooth angle.

## 1 Introduction

In pediatric dentistry, patients often present with chief complaints related to eruption disorders of permanent teeth. Among these, the abnormal eruption of the permanent central incisors is the most common. These teeth sometimes exhibit dilaceration and are impacted in an inverted position, precluding spontaneous eruption (1). Surgery or transalveolar autotransplantation of the impacted teeth has been reported in the literature (2–4). A longitudinal study of surgical repositioning revealed a high incidence of severe complications, which has limited the application of this approach for impacted teeth (5). Consequently, the two main treatment options for this condition are extraction and prosthetic replacement with a bridge or implant, or fenestration and orthodontic traction. In these cases, the factors that determine the treatment plan include the position and direction of the impacted tooth, degree of root formation and curvature, and availability of space for the impacted tooth (6, 7). Traction treatment is considered difficult for inversely impacted maxillary central incisors with a high crown axis inclination or root curvature. In such cases, tooth extraction is considered as a treatment strategy. Some orthodontists are reluctant to treat markedly dilacerated teeth, citing the potential for treatment failure secondary to complications such as ankylosis, loss of attachment, external root resorption, and root exposure following orthodontic traction (7–9). In instances of root exposure, endodontic treatment or apicoectomy may be necessary (7, 10). However, some case reports on orthodontic treatment that prioritize conservative treatment have been published (4, 11). Recently, more numbers of clinicians reported the success cases of severe dilacerated teeth (1, 12), however, most of reports are based on independent case report and the clear standard of choosing traction as treatment option is not well established. Based on these facts, the relationship between the possibility of success and the factors affecting the results of orthodontic treatment is important to examine by a case series. In this study, we present three cases of orthodontic treatment for dilaceration and inversely impacted maxillary central incisors and examine the timing of treatment, crown axis inclination, and root curvature of the central incisor. A coordinated multidisciplinary approach toward the management of impacted maxillary central incisors has resulted in favorable aesthetic and functional outcomes.

## 2 Materials and methods

**Figure 1:**
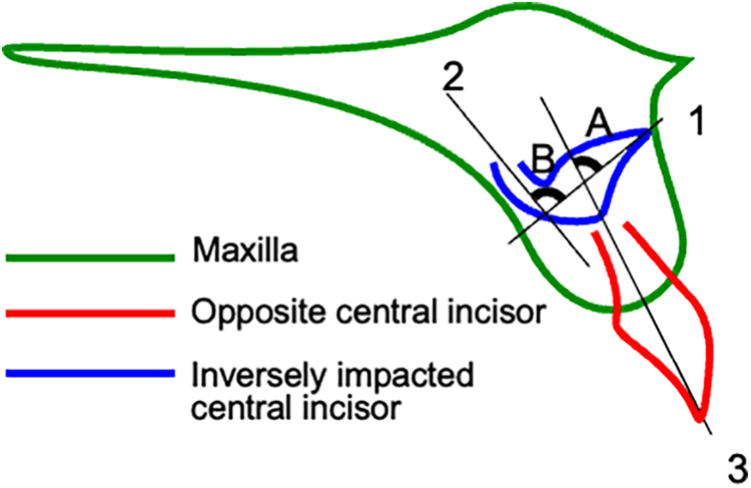
Tooth crown axis angulation, root curvature, and root formation measured using x-ray examination. Pre-treatment lateral cephalograms were obtained from the patients. The crown axis (1) and root axis (2) of the impacted central incisor and the tooth axis (3) of the opposing central incisor were plotted. The inclination angle of the crown axis **(A)** was measured using the crown axis of the impacted central incisor (line 1) and the tooth axis of the opposing central incisor (line 3). The angle of root curvature **(B)** was measured from the crown (line 1)-root (line 2) axis of the impacted central incisor.

The study included patients aged 7–10 years old who were diagnosed with inversely impacted maxillary central incisor and had visited the Department of Pediatric Dentistry at Tohoku University Hospital. The patients who have systemic diseases or mental retardation were excluded. The total subject number analyzed in this study is three (*n* = 3). Signed informed consent was obtained from the guardian of each study patient. The crown axis inclination, root curvature, and root formation were measured from the pre-treatment x-ray photographs of lateral cephalograms or computed tomography (CT) scans (Figure 1) (13). After the impacted central incisor was fenestrated, traction was applied using a lingual arch appliance with elastics, and finally the tooth was arranged using a multi-bracket orthodontic appliance. The duration of orthodontic treatment, crown axis inclination, root curvature, and root formation were analyzed in each case.

## 3 Results

### 3.1 Case 1: a girl aged 9 years and 1 month

**Figure 2:**
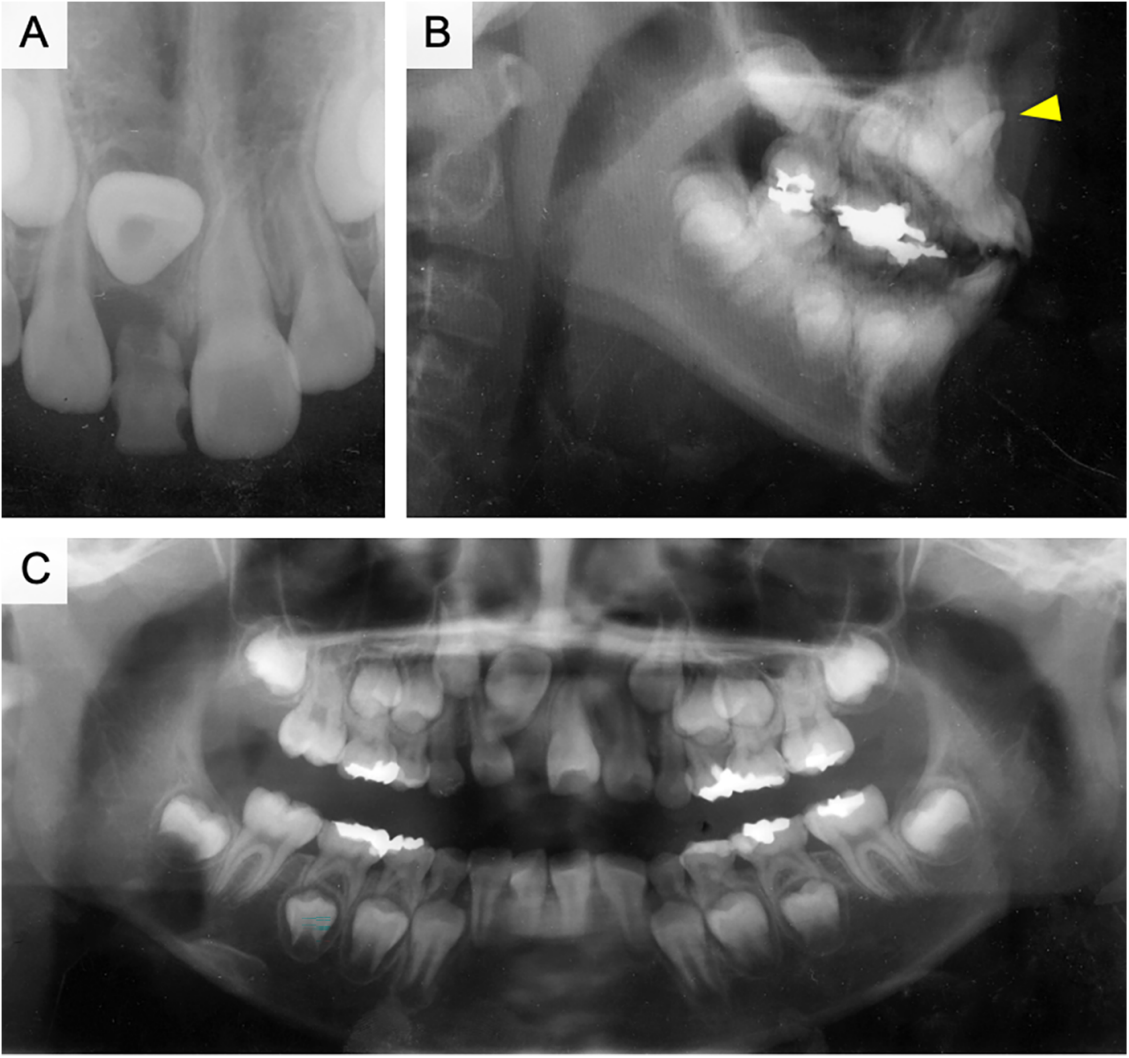
Pre-treatment x-ray photograph of case 1. **(A)** Dental x-ray photograph taken at the first consultation. **(B)** x-ray image of the sagittal plane. The red arrowhead is an inversely impacted maxillary central incisor. **(C)** Panoramic radiograph taken after the extraction of the upper right primary central incisor.

The patient visited our hospital for detailed examination and treatment of an impacted right maxillary central incisor. She underwent periodic examinations by the general dental practitioners (GDP), who indicated that the eruption of the right maxillary central incisor was delayed. The right maxillary lateral incisor had already erupted, whereas the right maxillary primary central incisor was still not exfoliated and insufficient eruption space was available for this tooth (Figure 2A). The right maxillary primary central incisor had composite resin restoration in medial and distal side, suggesting that there was dental caries. The history of dental trauma or intrusion was not clear. The mobility of right maxillary primary central incisor was moderate and its labial gingiva showed abscess. The root absorption of the right maxillary primary central incisor was 1/2, and periapical periodontitis was observed around the tooth root on radiographic examination. According to the radiographic findings, the right maxillary central incisor was turned upward and the root was curved toward the right lateral incisor (Figures 2B,C). The crown axis inclination and root curvature measured 120° and 110°, respectively, and the root formation was 1/2. The root formation of the left maxillary central incisor was almost complete. A slight delay in growth of the mandible was observed on cephalometric analysis (ANB = 6°). In addition, the upper and lower incisor angles were labially inclined (U1 to SN = 114°, L1 to Mand.Pl. = 102°). We analyzed the dental occlusal model and found that the anterior tooth occlusion was normal, the relationship between the left and right first molars was Class I, and lingual migration of the left maxillary lateral incisor was observed. The maxillary arch length discrepancy (ALD) was −1 mm.

**Figure 3:**
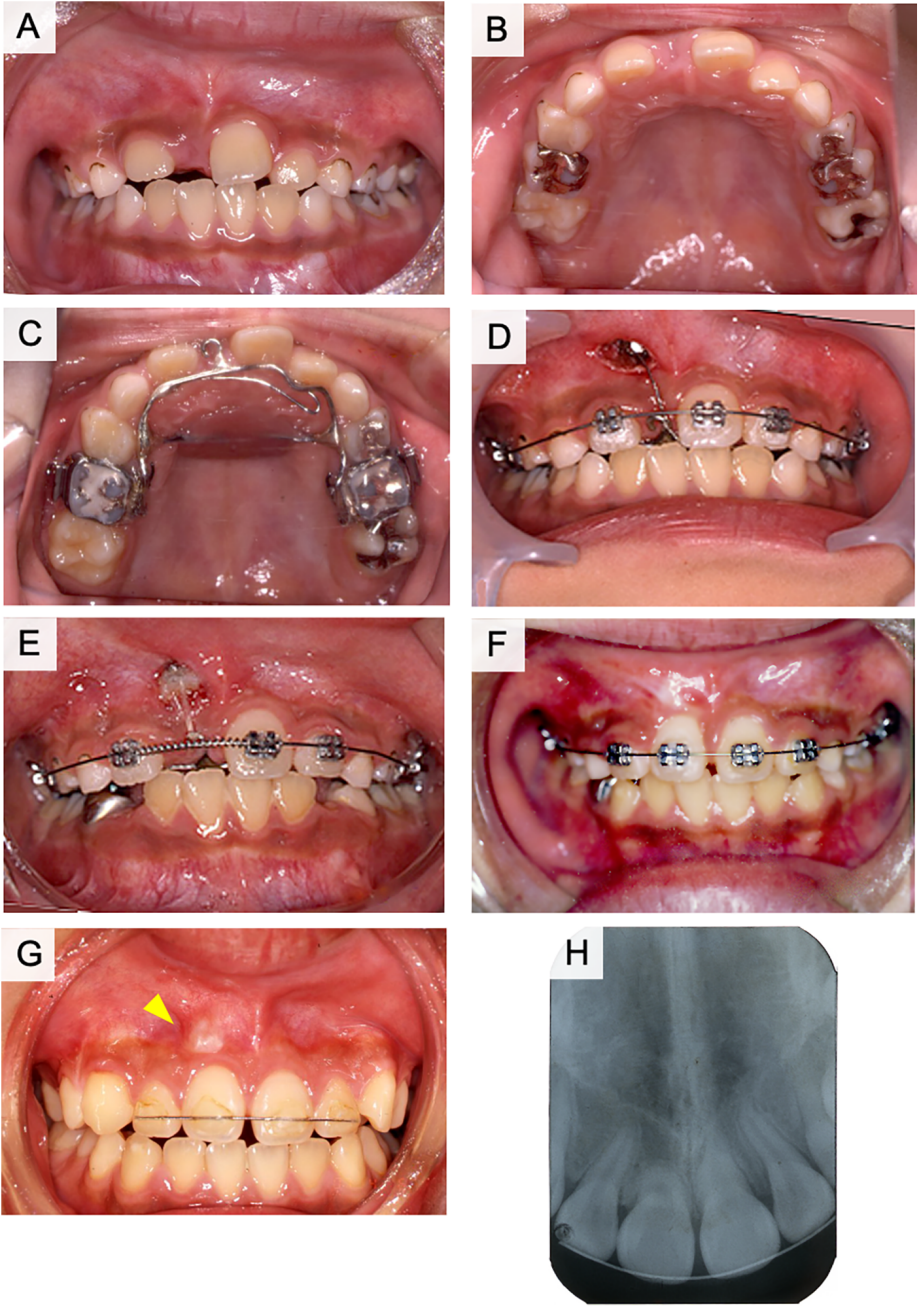
Treatment progress with fenestration and traction of the impacted tooth in case 1. **(A)** Frontal view of intraoral photograph after extraction of the upper right primary central incisor. **(B)** Maxillary occlusal image of intraoral photograph after extraction of the upper right primary central incisor. **(C)** Lingual arch appliance. **(D)** The start of the fenestration and traction. **(E)** Six months after the start of traction. **(F)** One year after the start of traction. **(G)** During the retention period. **(H)** Post-treatment x-ray photograph of case 1.

The right maxillary primary lateral incisor was extracted during the initial examination. However, the right upper central incisor had not erupted one month later (Figures 3A,B). In the next approach, we used a lingual arch appliance and a multi-bracket appliance on the upper jaw. The exposure and traction of dilacerated teeth has performed two months later from extraction of primary teeth. The eruption space was restored after orthodontic treatment (Figure 3C). Fenestration was performed, and a lingual button was attached to the lingual surface of the central incisor to provide traction after two months of treatment (Figure 3D). Six months after the beginning of traction, the lingual button was replaced on the labial side, and traction was continued (Figure 3E). The right maxillary central incisor was aligned with the dental arch after one year of treatment (Figure 3F). A bulge caused by the curved root apex was observed in the labial gingiva of the right maxillary incisor (Figure 3G, arrowhead). At this time, the root length of right maxillary central incisor was 1/2 compared to left maxillary incisor on radiographic examination (Figure 3H). Lamina dura is formed around the right maxillary central incisor and no radiolucent area was observed. The pulp of dilacerated right maxillary central incisor was vital after orthodontic treatment. During the following four years, the root apex of the right maxillary central incisor was located under the gingiva, and the pulp remained vital. The clinical crown length and gingival height was normal for the treated teeth.

### 3.2 Case 2: a boy aged 7 years and 5 months

**Figure 4:**
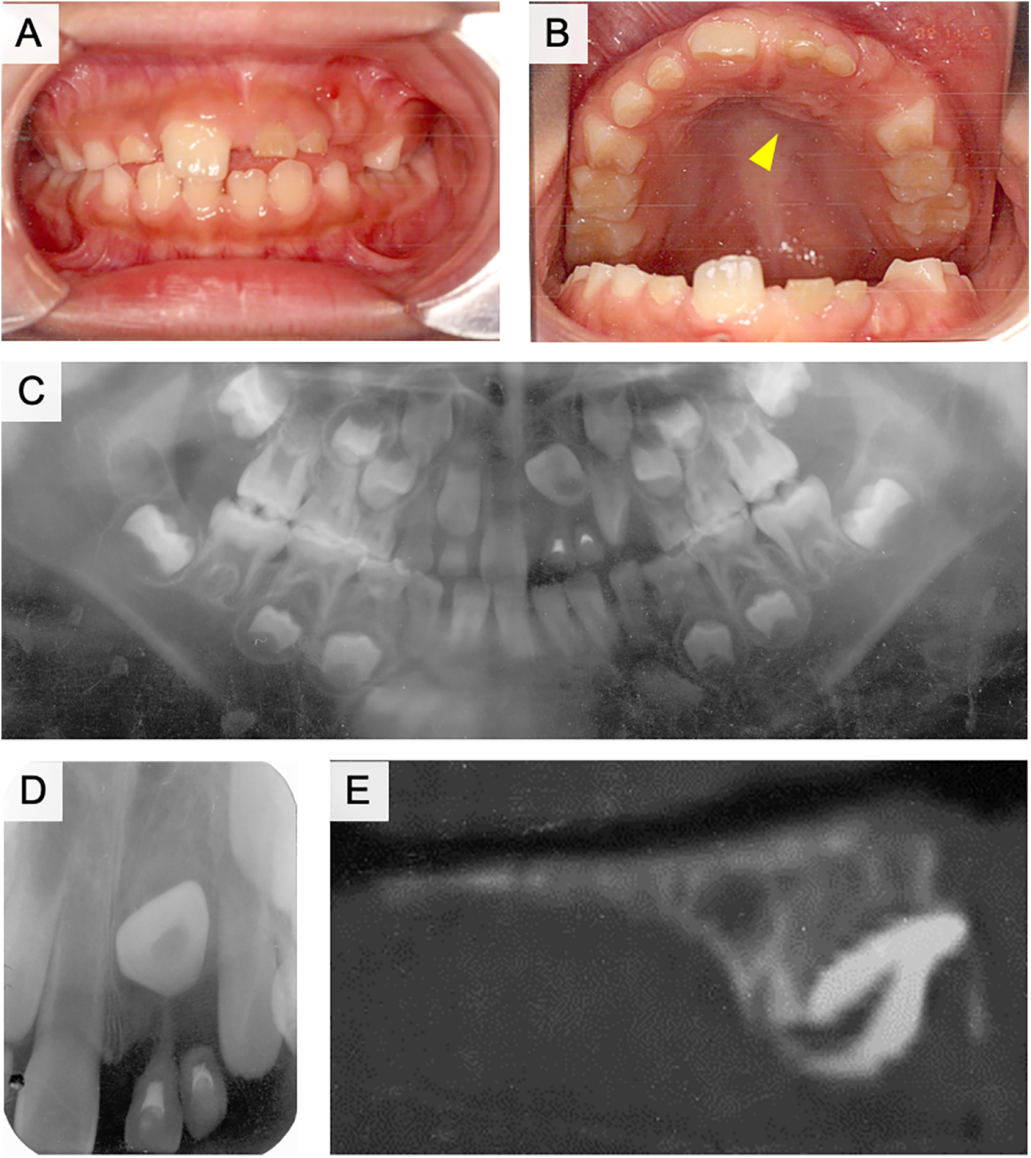
Intraoral and radiographic photographs at the initial examination of case 2. **(A)** Frontal view of intraoral photograph. **(B)** Maxillary occlusal view of intraoral photograph. **(C)** Panoramic radiograph. **(D)** Dental x-ray image of the left maxillary central incisor. **(E)** Sagittal plane image of the left maxillary central incisor using computed tomography (CT).

The patient visited our hospital with the main complaint of delayed eruption of the left maxillary central incisor. The right maxillary central incisor erupted at the age of 6 years, whereas the left maxillary central incisor had not yet erupted. A panoramic radiograph was obtained at the GDP clinic and showed that the left maxillary central incisor was impacted in an inverted position. The right maxillary central incisor had almost completely erupted; however, the left maxillary primary incisors were still present (Figures 4A,B). The left maxillary primary incisors were treated with vital pulpectomy and restored with composite resin in palatal side. The patient and parents did not clearly remember the history of dental trauma. The mobility of primary teeth was moderate and abscess was observed in the apical gingiva of left maxillary primary lateral incisor. A bulge was observed on the palatal side of the tooth, which appeared to be the root of the left maxillary central incisor (Figure 4B, arrowhead). The left maxillary primary canine was missing; however, the cause was unknown. Radiography results indicated that the left maxillary central incisor was impacted below the primary incisors, with the crown pointing upward (Figures 4C,D). The root of the left maxillary primary incisor was absorbed by external absorption from medial and distal side of root and a radiolucent area was observed in the alveolar bone around the roots (Figure 4D). The crown axis inclination and root curvature measured 110° and 112°, respectively, and the degree of root formation was 1/3 (Figure 4E). Analysis of the lateral cephalogram indicated that the angle was Class I and the intermaxillary relationship was characterized by ANB = 6.2°, although the maxilla and mandible were both slightly larger than normal in sagittal view (A’-Ptm’ = 46.1 > 1 S.D., Gn-Cd = 100.0 > 1 S.D.). The upper and lower central incisors showed a labial inclination (U1 to SN = 105°, L1 to Mand.Pl. = 93°). According to the analysis of the study model, the anterior tooth coverage was normal, the relationship between the left and right first molars was Class II, and the ALD of the upper and lower dental arches was positive.

**Figure 5:**
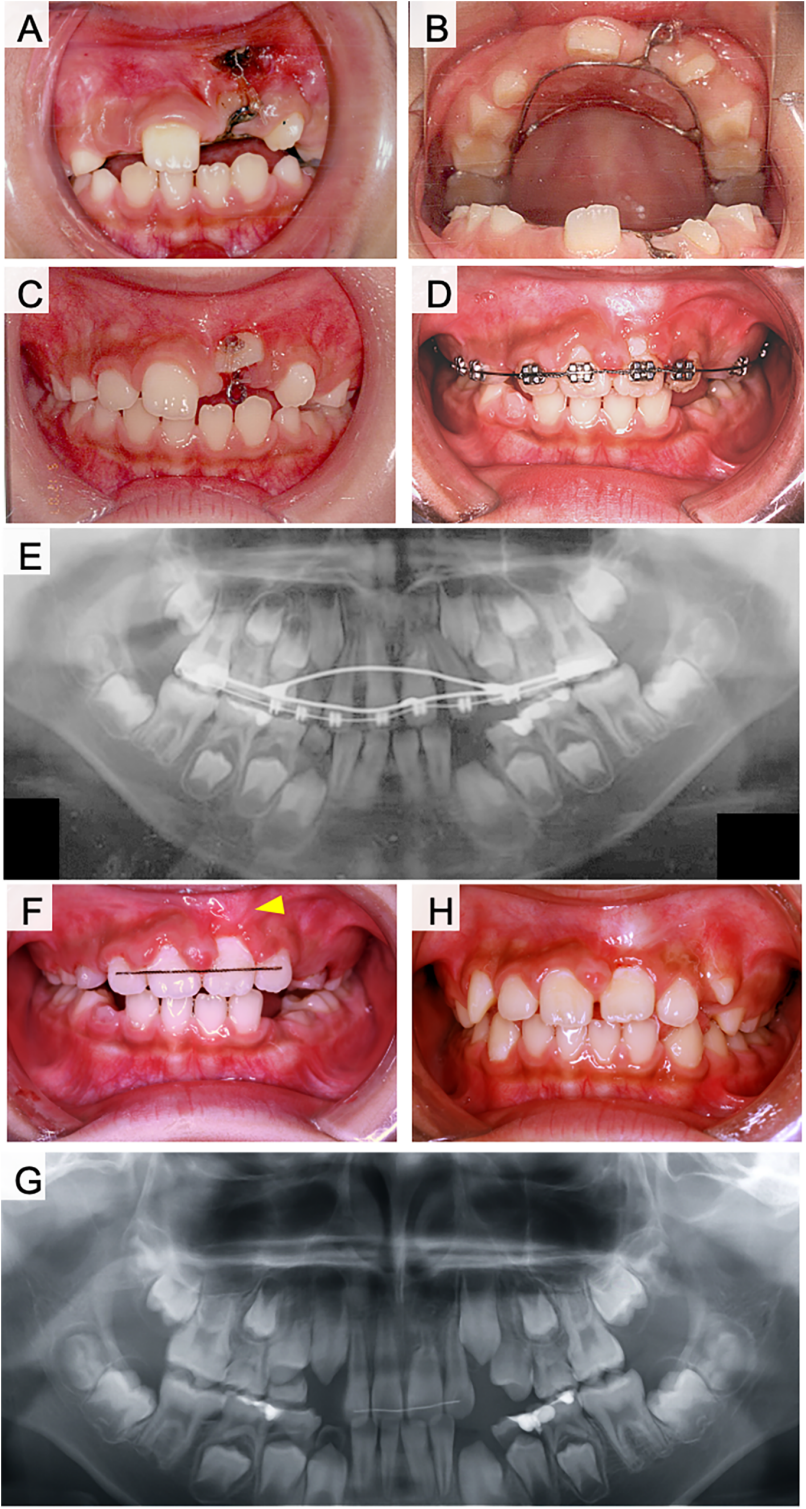
Treatment progress with fenestration and traction of the impacted tooth in case 2. **(A)** Frontal view of intraoral photograph at the start of the fenestration and traction. **(B)** Maxillary occlusal view of intraoral photograph at the start of the fenestration and traction. **(C)** Six months after the start of traction. **(D)** One year and five months after the start of traction. **(E)** Panoramic radiograph after one year and five months of traction. **(F)** During the retention period. **(G)** Panoramic radiograph of post-treatment. **(H)** One year and six months after the end of treatment.

The left maxillary primary incisors were extracted as a treatment strategy at first visit. A lingual arch was then applied four months later, and fenestration and traction of the left maxillary central incisor were initiated (Figures 5A,B). The incisal edge of the impacted central incisor was detected six months after the start of traction. Subsequently, the lingual button was replaced on the labial side and traction was continued (Figure 5C). After one year and five months of traction, the left maxillary central incisor was guided into the occlusion (Figure 5D). The bend in the root was observed at approximately 1/2 of the root length, and it was elongating upward (Figure 5E). Retention of the dental arch was then performed. The labial gingiva had receded, and the root curvature was detected under the labial alveolar mucosa (Figure 5F, arrowhead). At this time, root formation of the left maxillary central incisor was almost completed, while root curvature was observed around center of root length (Figure 5G). The root length of the left maxillary central incisor was 4/5 compared to the right maxillary central incisor. No external absorption of root was observed and the left maxillary central incisor was vital. Although a slight midline separation was observed, the left maxillary central incisor was vital after 1.5 years follow-up of treatment (Figure 5H). Crown height showed mild difference between left and right maxillary incisors, while alveolar bone level was similar.

### 3.3 Case 3: a girl aged 7 years and 8 months

**Figure 6:**
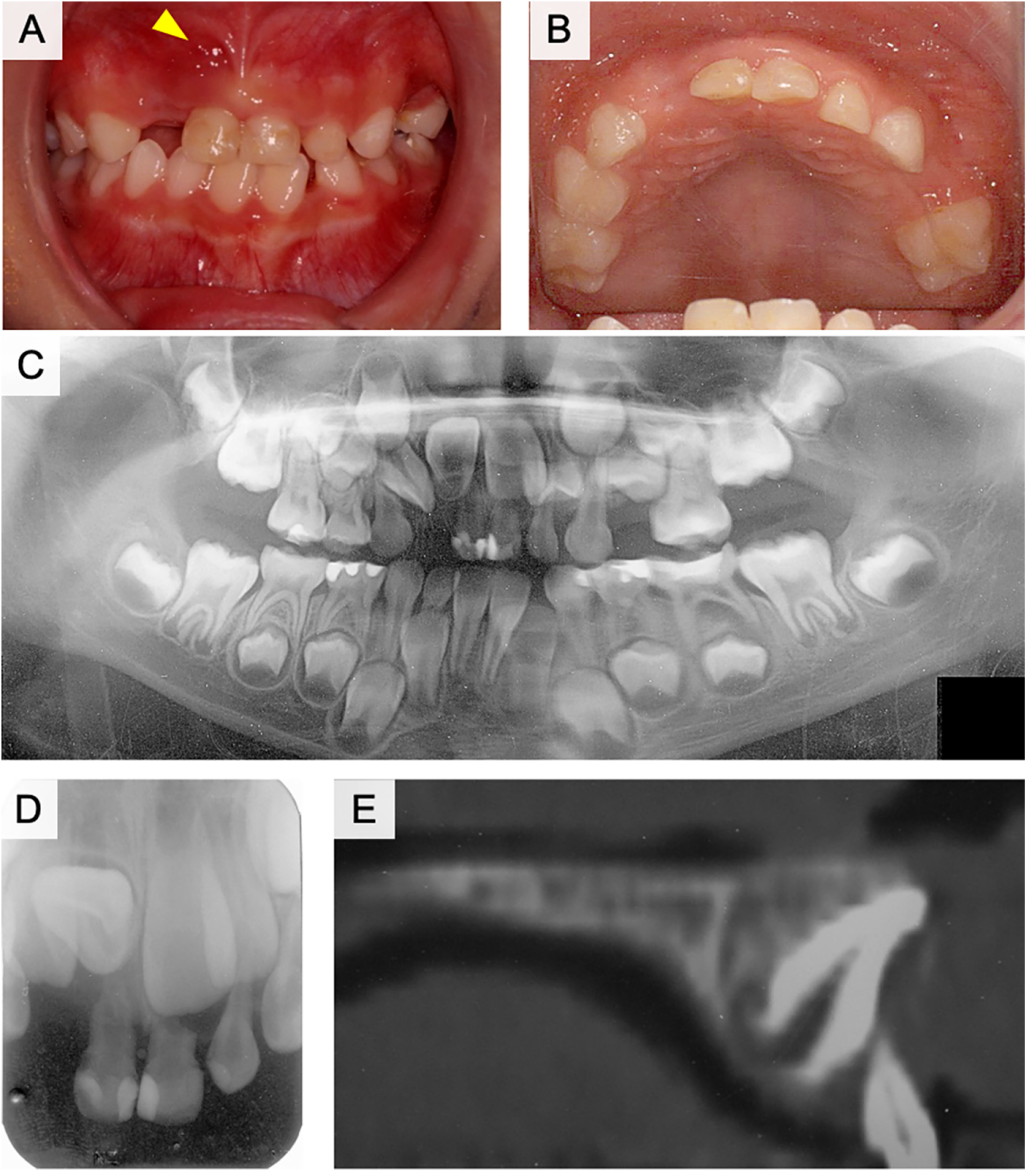
Intraoral and radiographic photographs at the initial examination of case 3. **(A)** Frontal view of intraoral photograph. **(B)** Maxillary occlusal view of intraoral photograph. **(C)** Panoramic radiograph. **(D)** Dental x-ray image of the right maxillary central incisor. **(E)** Sagittal plane image of the right maxillary central incisor using computed tomography (CT).

The patient presented to our hospital for the examination and treatment of an inversely impacted tooth on the right side of the maxilla. Dental radiographs were obtained at the GDP clinic for the diagnosis of caries and showed an abnormal direction of eruption of the right maxillary central incisor. The history of trauma was unclear during the interview. The left maxillary primary central incisor was filled with composite resin in mesial and distal side and showed moderate mobility because it was approaching dehiscence (Figures 6A,B). A bulge was observed at the gingivobuccal transition of the right maxillary primary central incisor, which appeared to be the incisal margin of a buried right maxillary central incisor (Figure 6A, arrowhead). Panoramic radiographs indicated that the crown of the right maxillary central incisor was turned upward and that the lateral incisor was twisted (Figure 6C). Dental radiographs demonstrated half resorption of the left maxillary primary central incisor, but not the root of the right primary central incisor (Figure 6D). The radiolucent area of the left maxillary primary central incisor was not clearly observed. The crown axis inclination and the root curvature measured 109° and 70°, respectively, and the degree of root formation was less than 1/4 (Figure 6E). Cephalometric and study model analyses were all within the normal ranges.

**Figure 7:**
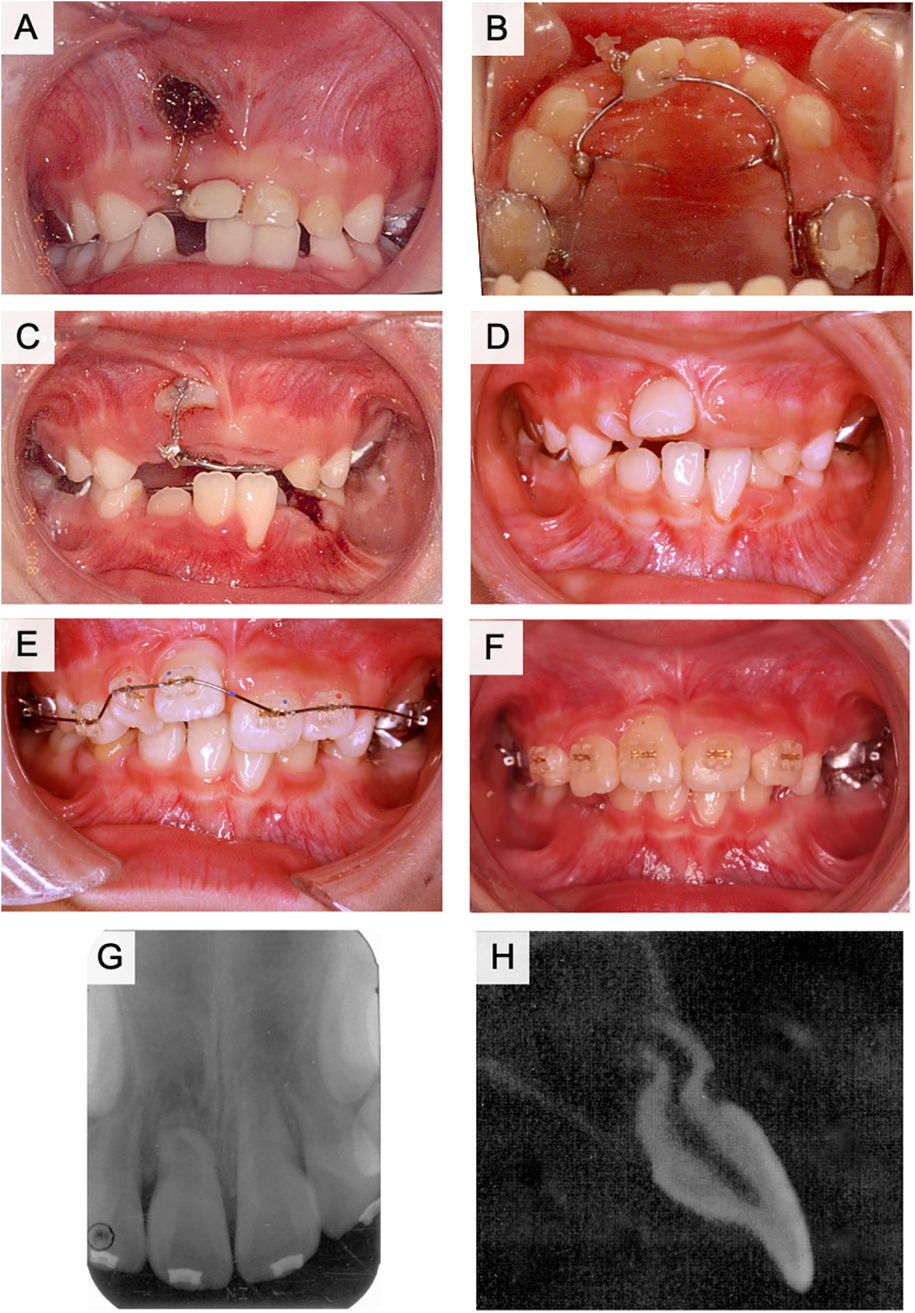
Treatment progress with fenestration and traction of the impacted tooth in case 3. **(A)** Frontal view of intraoral photograph at the start of the fenestration and traction. **(B)** Maxillary occlusal view of intraoral photograph at the start of the fenestration and traction. **(C)** Three months after the start of traction. **(D)** Eight months after the start of traction. **(E)** One year and six months after the start of traction. **(F)** Two years after the start of traction. **(G)** Dental x-ray photograph after two years of traction. **(H)** Computed tomography (CT) image of the right maxillary central incisor in the sagittal plane after two years of traction.

The right maxillary lateral deciduous incisor was extracted, and two months later a lingual arch appliance was used for the maxilla, and fenestration and traction of the right maxillary central incisor were initiated (Figures 7A,B). The right maxillary central incisor was in a horizontal position three months after the start of traction. Thereafter, the lingual button was replaced on the labial side and traction was continued (Figure 7C). After eight months of traction, the tooth axis improved (Figure 7D), and the teeth were aligned using a multi-bracket system after the eruption of the maxillary incisors (Figure 7E). The right maxillary central incisor was guided into the dentition two years after the start of traction, although some retraction of the labial gingiva was observed (Figure 7F). Radiographically, the root of the right maxillary central incisor was curved in a sigmoidal shape (Figures 7G,H). No radiolucent area was observed around the root of the right maxillary central incisor. Although alveolar bone level did not show abnormality, the shape of gingival margin was asymmetry between left and right incisors.

### 3.4 The characteristics of inversely impacted teeth in all cases

The average age of the three patients was 8 years and 1 month (Table 1). The crown axis inclination was 113°and was more inverted than horizontal. The degree of dentin curvature was 97.3°, which was more curved than perpendicular. The degree of root formation was 36.1%, which was less than 1/2 root formation. The average treatment period was one year and six months.

**Table 1:** Condition of the maxillary central incisor in each case and duration of treatment.

Subject	Years	Crown axis inclination	Root curvature	Root formation	Treatment period
Case 1	9 years 1 month	120°	110°	50%	1 year 1 month
Case 2	7 years 5 months	110°	112°	33.3%	1 year 5 months
Case 3	7 years 8 months	109°	70°	25%	2 years 0 months
Average	8 years 1 month	113°	97.3°	36.1%	1 year 6 months

## 4 Discussion

Dilaceration may affect both permanent and primary teeth, but dilaceration of the primary teeth is rare (14). The prevalence in maxillary central and lateral incisors is 0.4%–1.2%, respectively (15). Dilaceration of the crown of a permanent tooth is responsible for 3% of all traumatic injuries to developing teeth and is usually a result of the intrusion or avulsion of its primary predecessor (16). The stage of development at which the injury occurs determines the clinical presentation of dilaceration in the succeeding permanent tooth (17). The injured Hertwig's epithelial root sheath continues to produce dentin at the same rate as before the injury. Therefore, the final root shape of the permanent maxillary central incisor forms in a continuous labial curve until apex formation is complete (18). Furthermore, the Hertwig's epithelial root sheath remains in place within the alveolar process against the eruptive forces of the developing tooth and guides the orientation of root development. Consequently, the crown of the permanent central incisor appears to move labially and upward as long as the asymmetric calcification of the root continues (18). In cases 1 and 2, radiolucency was observed around the root of the preceding primary tooth, suggesting the presence of a root lesion of the primary central incisor with inflammation around the root area, which caused the dilaceration of the permanent teeth. In case 2, both the left primary central and primary lateral incisors were non-vital, and the left primary canine was missing at the first visit. These dental findings indicate the possibility of a history of trauma which may have affected the dilaceration. In case 3, no periapical disease was observed in the right primary central incisor, and the history of trauma was unclear during the interview. A right maxillary lateral primary incisor was extracted by the GDP because of significant tooth movement; however, the cause of this movement was not identified. Therefore, periapical disease was suspected.

Several treatment plans are considered for dilacerated teeth: (i) surgically extracting the dilacerated teeth and maintaining the extraction space for restoration with a bridge or an implant. (ii) surgically extracting the dilacerated teeth and orthodontically closing the extraction space and prosthodontic restoration of the lateral incisor. (iii) *in situ* rotation surgery for correction of the dilacerated teeth. (iv) orthodontic traction of dilacerated teeth into proper alignment with or without orthodontic space opening (19). Orthodontic traction has various advantages as preservation of the patient's natural teeth, prevention of future restorations or implants, maintenance of the height and width of the alveolar bone in the anterior tooth region, and improvement of esthetic outcomes (12). In this study, we presented these treatment options to the patients and their parents and we selected orthodontic traction for the first choice of treatment plan. Although all our cases successfully aligned the dilacerated teeth into proper dental arch with vital pulp, the traction of severely dilacerated teeth is challenging. It is necessary to explain the patients that during traction pulp may result in non-vital and may require apicoectomy, and failure of traction may lead to choose extraction of dilacerated teeth for further treatment (7). Our cases remained vital teeth during treatment, however, labial bulge was observed in some case. Long-term follow-up of the vitality of pulp is necessary after traction of dilacerated teeth. The present cases used open-exposure technique for its advantages in operability of traction. While, some difference in margin of gingiva was observed after orthodontic alignment of dilacerated teeth into dental arch. Although these gingiva shape may change as symmetry by growth of alveolar bone, the closed-exposure technique may result in better prognosis of aesthetics (18).

For the orthodontic traction of dilacerated teeth, the following factors are reported to be important for good prognosis: (i) the position of impacted teeth, (ii) the degree of root formation, (iii) inclination angle of root and coronal axis (4, 6, 7, 20, 21). Traction of dilacerated teeth is considered as a treatment option when the root inclination angle is less than 90° (15). In some cases, rotational surgery is performed to correct the angle (19, 22, 23). However, intentional replantation of dilacerated teeth is not recommended because extraction of the dilacerated tooth may be difficult and cause fracture of the dilacerated root (24). Root dilaceration concentrates stress in the supporting structures when a dilacerated tooth is used as an abutment for a dental prosthesis. Therefore, as an abutment tooth, a dilacerated tooth has a high risk of fracture. Alternatively, splinting of the dilacerated abutment tooth may be an approach that should be considered in some cases (25). These approaches are options for improving orthodontic treatment (26–29). Crown height, crown-root angle, and patient age are considered important factors influencing the duration of orthodontic treatment (20, 30, 31). Cases of failure of traction of dilacerated teeth are often related to the following factors: older age, male sex, higher maxillary positioned impacted teeth, abnormal root resorption, and failure to improve position by ankylosis (32, 33). In this study, case 1 had a coronal axis inclination of 120° and a root curvature of 110°, case 2 had a coronal axis inclination of 110° and a root curvature of 112°, and case 3 had a coronal axis inclination of 109° and a root curvature of 70° (Table 1). Although traction was expected to be difficult in these cases, fenestration and traction were selected instead of extraction. As a result, the impacted dilaceration was successfully guided into the dentition in all three cases. In cases 1 and 2, the flexed root was palpable sublingually (Figures 3F, 5F, arrowheads). In case 3, the root did not detect the mucosa but was curved in a sigmoidal shape. The pulp of the dilacerated tooth was vital after treatment in all cases. Case 1 had 1/2 the amount of root formation, case 2 had 1/3, and case 3 had less than 1/4 at the starting point of traction. All the patients had incomplete roots (Table 1). Shi et al. proposed the traction of dilacerated teeth results in good prognosis when the traction starts in younger age, especially under 10 years-old (21). The present cases started traction of dilacerated teeth under 10 years-old. Furthermore, traction prognosis is considered relatively good for teeth with incomplete roots because of abundant blood flow and active alveolar bone formation (4, 34). In cases 2 and 3, root growth was not arrested by traction treatment, and the roots were elongated despite flexion. In addition to pulp canal obliteration, yellow or gray discoloration and pulp necrosis may also be observed in traction-treated dilacerations (35). The root curvature in case 3 was probably influenced by the large crown axis inclination (109°), despite the small amount of root formation. Therefore, the degree of root curvature at the start of traction may be influenced by the degree of crown axis inclination and the amount of root formation. The degree of crown axis inclination and amount of root formation at the start of traction are important factors that determine the success or failure of traction (4, 6, 7, 21).

In all three cases, the button was attached to the lingual surface of the fenestrated tooth, and traction was initiated using a lingual arch appliance and elastics. The treatment period with the lingual button approach was six months for case 1, eight months for case 2, and three months for case 3, before button replacement to the labial surface. After improvement of the crown axis, a duration of one year for case 1 and one year and five months for case 2 was required to align with the dental arch. Case 3 required two years for alignment because there was an observation period for the incisor eruption. A previous study reported that the average duration of treatment was 21.6 ± 8.7 months and was correlated with tooth height (32). Another study reported nine months of traction and an overall treatment duration of 21 months (32). Therefore, traction speed was appropriate in these cases.

These results suggest that the key factor in deciding the treatment strategy for an inverted maxillary central incisor is not the difficulty of traction but rather the ability of the tooth to function after treatment. The determining factors may be the degree of root curvature and depth. Therefore, if a tooth is diagnosed with dilaceration, treatment should be started before the root becomes curved and does not move to a higher position. Therefore, early detection is important, especially in cases where the preceding primary tooth has a history of trauma or endodontic treatment. Considering the possibility of an abnormal eruption of the permanent incisor, the incisor should be carefully observed from approximately 6 years of age. If a left-right difference in the eruption timing of the permanent teeth is observed, an abnormal eruption direction should be suspected, and the tooth should be examined using radiography.

The limitation of this study is considered as following: (i) the sample size was small and limited in the distribution of patient's age. (ii) We performed the cases which the root formation of dilacerated teeth was not completed, therefore it is necessary to compare the success rate with the cases that root formation is completed. Further analysis is required for gathering more numbers of cases in future.

## 5 Conclusion

In these cases, the tooth that was the reverse of the maxillary implantation was aligned using orthodontic treatment. All dilacerations were vital and root formation was continued, although in some cases, a bulge was observed in the gingiva on the labial side of the root apex region of the incisor after treatment. In addition, the early treatment could fix the root elongation axis. These findings suggest that early traction of dilaceration leads to good prognosis. When an inversely impacted incisor is identified, traction should be performed as early as possible before root formation progresses, without considering the degree of inclination of the crown axis or the degree of root curvature.

## Data Availability

The original contributions presented in the study are included in the article/Supplementary Material, further inquiries can be directed to the corresponding author.
